# Study protocol on effectiveness of yoga practice on composite biomarker age predictors (yBioAge) in an elderly Indian cohort- two-armed open label randomized controlled trial

**DOI:** 10.1186/s12877-023-04517-6

**Published:** 2023-12-15

**Authors:** Vijaya Majumdar, N. K. Manjunath, Atmakur Snigdha, Prosenjeet Chakraborty, Robin Majumdar

**Affiliations:** 1 Division of Life Science, Molecular Bioscience Lab, Swami Vivekananda Yoga Anusandhana Samsthana, Bangalore, Karnataka 560105 India; 2 Indian Institute of Information Technology, Bangalore, Karnataka 560100 India

**Keywords:** Biological age, Yoga, Aging

## Abstract

**Introduction:**

The recent development of robust indices to quantify biological aging, along with the dynamic epidemiological transitions of population aging generate the unmet need to examine the extent up to which potential interventions can delay, halt or temporarily modulate aging trajectories.

**Methods and analysis:**

The study is a two-armed, open label randomised controlled trial. We aim to recruit 166 subjects, aged 60–75 years from the residential communities and old age clubs in Bangalore city, India, who will undergo randomisation into intervention or control arms (1:1). Intervention will include yoga sessions tailored for the older adults, 1 h per day for 5 days a week, spread for 12 months. Data would be collected at the baseline, 26^th^ week and 52^nd^ week. The primary outcome of the study is estimation in biological age with yoga practice. The secondary outcomes will include cardinal mechanistic indicators of aging- telomere length, interleukin-6 (IL-6), tumor necrosis factor receptor II (TNF-RII), high sensitivity c-reactive protein (hsCRP)], insulin signaling [insulin and IGF1], renal function [cystatin], senescence [growth differentiating factor 15 (GDF-15)] and cardiovascular function [N-terminal B-type natriuretic peptides (NT-proBNP)]**.** Analyses will be by intention-to-treat model.

**Ethics & dissemination:**

The study is approved by the Institutional Ethics Committee of Swami Vivekananda Yoga Anusandhana Samsthana University, Bangalore (ID:RES/IEC-SVYASA/242/2022). Written informed consent will be obtained from each participant prior to inclusion.

**Trial registration number:**

CTRI/2022/07/044442.

## Introduction 

Global ageing, paralleled with increasing mental, behavioural and cardiovascular health morbidities, elicits many public health concerns. This demographic transition has been estimated to lead to a worldwide increase in the prevalence of older adults aged ≥ 60 years by 500 million over 15 years [from 900 million in 2015 to 1400 million by 2030] [1, 2]. In India, older adults constitute 8.6% of the total population as per Census 2011, projected to reach 19% by 2050 [3]. Unfortunately, the increase in lifespan has not been paralleled with an equivalent increase in healthy life expectancy or health span [4, 5]. Hence, developing and implementing anti-ageing therapies or interventions that could "healthy" life span has received significant scientific attention [5, 6]. The pre-clinical experimental evidence supports the feasibility of interventions for extending healthy life span in model organisms. However, in humans, the lengthy lifespan and the delayed time course for the symptomatic manifestation of ageing-related health decline pose barriers to clinical translation. To this end, a quest has been to develop robust markers or indices that could quantify the process of biological ageing, termed as the "BA estimates" [6]. Based on the complexity of the ageing process in humans, reliance on a single-point measurement has been considered a limitation [4, 5]. Though several biomarkers mimic age-related physiological deregulation at individual levels, composite predictors derived from merging multiple biomarkers into single latent variables has been considered a better approach to capture the complex aetiology of the ageing process. Developing these quantifiable indices of ageing is considered as the most critical milestone of geriatric research to aid in the longitudinal tracking of the trajectory of physiological decline. Klemera and Doubal-based Biological Age (KDM-BA) is such an algorithm-based measure that represents the status of integrity of multiple organ systems in the body [7]. KDM-BA is derived from a panel of various biomarkers measuring system integrity, including cardiovascular, renal, hepatic, immune, and metabolic function: albumin, blood urea nitrogen, creatinine, C-reactive protein, cytomegalovirus optical density, glycated haemoglobin total cholesterol, white blood cell count, lymphocyte per cent, mean corpuscular volume, systolic blood pressure, low-density lipoprotein- cholesterol. Based on the National Health Insurance Service-National Sample Cohort (NHIS-NSC) database, KDM-BA predicts morbidity, mortality, indicators of health span and age-related disease incidence in different populations as reported [7].

Several promising strategies have been posited as potential geriatric interventions in humans for their effects on health span. Lifestyle interventions (physical activity, calorie restriction, etc.) have restored immune functioning in aged individuals [8–12]. Dietary interventions included periodic fasting mimicking diets, protein restriction, drugs that inhibit the growth hormone/IGF-I axis, the mTOR–S6K pathway, or AMPK/sirtuins-based drug targets [13]. To this end, a recently reported secondary analysis of the landmark clinical trial “CALERIE” [14] indicates the potential of lifestyle interventions like calorie restriction in harnessing the process of biological ageing in humans with favourable changes in biomarkers related to cardiovascular and glucoregulatory functions. Yoga is a mind–body lifestyle modification technique, an amalgamation of physical activity, meditation and breathing techniques. A broad spectrum of health benefits for older adults has been reported to be associated with yoga [15–23]. However, most of the reported studies on yoga have targeted mobility, flexibility, cognition, gait, frailty, self-rated health and well-being as the outcomes [24–27]. Several preliminary reports also support the anti-ageing effects of yoga via key hallmarks of biological ageing such as telomere, attrition, regulation of inflammation and genome instability via DNA damage [28, 29].

There have been mechanistic postulates as well supported by molecular evidence base to support these beneficial aspects of yoga as an intervention, particularly for organ-specific or cellular functions. These insights could form an overarching framework for the exploration of the cumulative benefits of yoga in aiding physiological integrity. Hence, we aim to test the effectiveness of yoga in improving the multisystem decline in physiologic markers using KDM-based BA estimates.

Though the predictability of physiological status is well established through KDM-based age predictors, these predictors do not include several cardinal mechanistic biomarkers underlying the ageing process. Hence, we also propose to include a secondary list of blood-based biomarkers adapted from the study “Targeting Aging with Metformin (TAME) [5]. The TAME study includes a panel of outcome variables proposed to be used for geriatric intervention trials targeting healthy lifespan [5], including cardinal markers like telomere length, inflammation [interleukin-6 (IL-6), tumour necrosis factor receptor II (TNF RII), high sensitivity c-reactive protein (hs-CRP)], insulin signalling [insulin and IGF1], renal function [cystatin], senescence [growth differentiating factor 15 (GDF-15)] and cardiovascular function [N-terminal B-type natriuretic peptides (NT-proBNP)].

## Methods and analysis

### Study design

**Fig. 1:**
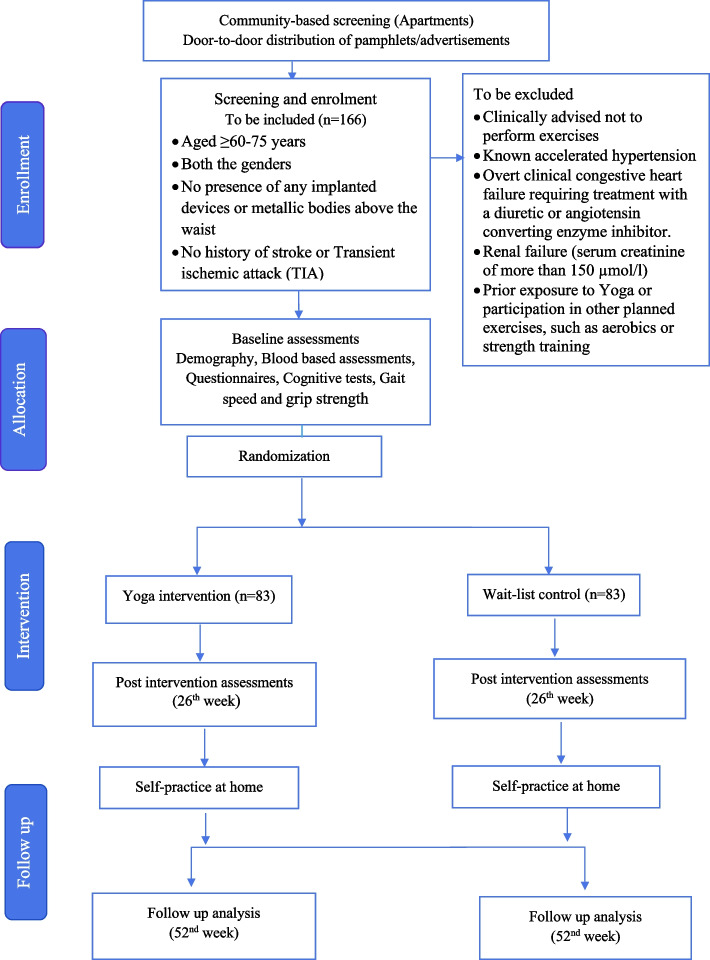
Trial profile

The study, yBioAge, is an open-label, two-arm, parallel-group, randomized, outcome-assessor blinded, clinical trial. The protocol has been drafted following the Consolidated Standards of Reporting Trials (CONSORT) guidelines [30] (Fig. 1). We plan to enrol 166 subjects across various sites in Bangalore, India. Detailed study information will be provided to all individuals while screening for eligibility criteria. Individuals not fitting into the eligibility criteria will be excluded from participation. The inclusion criteria are as follows: age ≥ 60–75 years, both genders, willingness to participate in the trial, and no intention to remain in the local area for the intervention or testing period. Exclusion criteria include individuals clinically advised not to perform exercises, those with prior exposure to Yoga, or involved in current or recent (last two months) practice of Yoga or with regular (three times per week or more) participation in other planned exercises, such as aerobics or strength training, individuals with known accelerated hypertension or/and uncontrolled diabetes or overt clinical congestive heart failure requiring treatment with a diuretic or angiotensin-converting enzyme inhibitor or with renal failure (serum creatinine of more than 150 µmol/l); individuals with any condition/s expected to severely limit survival, e.g., terminal illness; those with history of stroke or transient ischemic attack, or clinical diagnosis of dementia, inability to stand up or walk, participation in a drug trial within the past month preceding selection, alcohol or drug abuse, and presence of an implanted devices or metallic bodies above the waist. Interested eligible subjects will obtain written informed consent. Following the same, eligible subjects will be randomly allocated to yoga intervention or waitlist control arms. Intervention would be provided for six months and an end-point assessment will be after one year.

### Randomisation and blinding

An external statistician will generate a randomization list using a sequence randomizer. The list will be transferred into separate sealed envelopes and opened by research assistants and the participant immediately following the baseline assessment. As complete blinding is not possible due to the nature of the intervention to reduce the detection bias, investigators involved in evaluating outcome measures would be blinded for the randomization groups.

### Intervention

Participants randomized to the yoga group will receive a yoga module that has been tested for efficacy, safety and feasibility for older individuals [31]. The module effectively improves the quality of life and sleep in elderly individuals [32] and will comprise of a combination of yoga postures, breathing practices, meditative techniques, and relaxation. The physical postures will consist of loosening and stretching exercises followed by practice of physical postures (a*sana)* in standing, sitting, prone, and supine positions followed by *pranayama*. It provides a moderate intensity of physical activity. The patients will be instructed to follow a specific breathing pattern, culminating in a relaxation response during the practice. The practices will be interspersed by relaxation techniques to avoid any strain or exhaustion to the participants. Certified yoga instructors experienced in teaching older adults will deliver yoga classes in groups. The 60-min classes will be offered at multiple locations 5 days a week. The groups would comprise 20–25 participants, adequate to provide individual attention to each participant. Overall, the yoga intervention will.

be divided into 2 phases: phase 1 including 120 supervised sessions at the centres; phase 2 including at-home yoga practice sessions for next 6 months (24 weeks) (Fig. 1).

### Waitlist group

We decided on a waitlist design for the inactive control group because we deemed it ethically acceptable alternative to continuing to give the inactive older persons with the care they need beyond the trial. They will be told to carry on with their regular daily activities (without participating in scheduled, routine exercise) while being recruited. These participants will receive the same yoga-based lifestyle intervention offered to the intervention group following the completion of the 12 months study.

### Study outcomes

The primary outcome of the study is the change in scores of the KDM-based biological Age (BA) predictors from baseline to follow-up at 52 weeks (Table 1). For the KDM multiple candidate biomarkers based on their relevance in the aging process [7]. Effects of Yoga will also be assessed on several other cardinal mechanistic indicators of aging, markers of inflammaging [IL-6, hsCRP and TNFα], insulin signalling [insulin and IGF-1], renal function [cystatin], senescence [GDF-1], and cardiovascular function [NT-proBNP] [5]. Detailed explanation of secondary markers is given in Table 2.

**Table 1:** Primary outcome- biological age

KDM- BA panel	Glycated haemoglobin (HbA1c), Forced expiratory volume in one second (FEV1), Systolic Blood pressure (SBP), Total cholesterol, High sensitive C-reactive protein (hsCRP), Creatinine, Urea nitrogen, Albumin, Alkaline phosphatase (ALP), Cytomegalovirus IgG (CMV IgG), Fasting blood sugar (FBS), Low density lipoprotein – Cholesterol (LDL-c)

*Abbreviations*: *BA* Biological age, *KDM* Klemera–Doubal method based

**Table 2:** Secondary outcome panel

Mechanistic Bio markers	IL-6, hs-CRP and Tumour necrosis factor receptor II
	Insulin signaling (insulin and IGF-1)
	Renal function (cystatin)
	Senescence (GDF-15)
	Cardiovascular function (NTproBNP)
Body Composition	Body mass index (BMI)
Aging marker	Telomere length
Strength	Hand grip strength
Locomotion	Gait speed
Balance	Berg balance scale
Cognition	Digit symbol substitution task
Functionality	Activities of daily living

*Abbreviations*: *IL-6* interleukin-6, *hsCRP* high-sensitivity C-reactive protein, *TNF* alpha- Tumour Necrosis Factor alpha, *IGF-1* Insulin-like growth factor 1, *GDF-15* Growth differentiation factor 15, *NTproBNP N*-terminal proBNP, *BNP* Brain natriuretic peptide

### Assessments

The schedule of enrolment, interventions and assessments has been reported according to Standard Protocol Items: Recommendations for Interventional Trials 2013 guidelines in in Table 3. Given the nature of yoga intervention, we cannot do blinding of the participants to the intervention. However, to reduce the potential detection bias, the outcome assessors will be blinded to intervention allocations. An assigned research staff will be performing the assessments and he/she will be blinded to the subject allocation**.** The baseline assessment will be performed before randomisation. The assessment covers basic demographic questionnaire including age and date of birth, sex, marital status, occupation, income, education, co morbidities and medications currently in use will be obtained from each participant. Anthropometric measurements like height, weight, BMI and waist to hip ratio will also be obtained. Alcohol or drug abuse is termed as consumption of 5 plus drinks on one or more occasions during the prior year as an indicator of risky drinking behaviour [33]. Venepuncture will be done post a minimum of 8-h fasting, and blood samples will be collected in EDTA tubes.

**Table 3:** Schedule of enrolment, interventions and assessments, according to SPIRIT 2013 guidelines

**Study period**						
**Time points**		Enrolment	Baseline	Intervention	Post intervention	Follow –up
		Week -1	Week 0	Week 1–26	Week 26 + 2	Week 52 + 2
Eligibility screening		x				
Informed consent		x				
Allocation		x				
Intervention				x		
Yoga				x		
Waitlist					x	x
Assessments						
Demographics	Study Proforma		x			
KDM predictors of biological age	Glycated hemoglobin, Forced expiratory volume in one second, Systolic Blood pressure, Total cholesterol, C-reactive protein, Creatinine, Urea nitrogen, Albumin, Alkaline phosphatase, fasting blood sugar, low density lipoprotein- Cholesterol and Cytomegalovirus IgG		x		x	x
Aging marker	Telomere length		x		x	x
Body Composition	Body mass index (BMI)		x		x	x
Mechanistic markers	Inflammation [IL-6, hsCRP, TNF-RII]		x		x	x
	Insulin signaling [Insulin and IGF1]		x		x	x
	Renal function (cystatin)		x		x	x
	Senescence [GDF-15]		x		x	x
	Cardiovascular function [NT proBNP]		x		x	x
Strength	Hand grip strength		x		x	x
Locomotion	Gait speed					
Balance	Berg balance scale		x		x	x
Cognition	Digit symbol substitution task		x		x	x
Functionality	Activities of daily living		x		x	x

### Measuring biological age

Biological Age (BA) will be estimated using the Klemera–Doubal method (KDM) that combines information from multiple clinical biomarkers to quantify aging-related deficits in system integrity [7]. The KDM consists of computation of biological ages from multiple predictors (Glycated haemoglobin (Hba1c) will be measured by using the Beckman Coulter Chemistry Analyser AU480 system according to the manufacturer’s guidelines. A portable spirometer (Schiller spirometer (SP-1A) will be used to test lung function of the participants. Forceful exhalation for 1 s after a maximum inhalation will be set as forced expiratory volume in 1 s (FEV1), Digital BP monitor Omron model HEM-7120 (Omron Healthcare Co. Ltd.) will be used for measurement of Blood pressure at the end of the 5 min of rest as the average of two measurements 60 s apart. Low density lipoprotein-cholesterol (LDL-c), Complete hemogram, Urea nitrogen, Serum creatinine, Total cholesterol, Albumin, Alkaline phosphatase will be assessed by biochemical analyser (Clinical Chemistry Analysers—RX series-Randox), High sensitive C-reactive protein (hsCRP), and Cytomegalovirus IgG (CMV IgG) will be assessed through enzyme linked immunosorbent assay (ELISA- R&D systems) using the linear relationship between predictor and chronological age as classical calibration. We will use the BioAge R package 2 and PhenoAge algorithms first with custom sets of biomarkers, followed by projecting these algorithms onto our datasets [34]. The final product will be KDM-BA in unit of years. To account for the effect of CA, KDM-BA acceleration (KDM-BA acc), defined as the residual resulting from a linear model when regressing KDM-BA on CA will also be calculated.

### Telomere length

For estimation of telomere length, we will use a quantitative polymerase chain reaction (qPCR)-based method [35], with β-haemoglobin (Hbg) as a single copy reference gene. Relative TL of the target sample to the reference sample will be calculated according to the comparative 2 − ΔΔCq (Quantification Cycle Value) method.: relative T/S ratio = 2-ΔΔCt where ΔΔCt = (Ct Telomere − Ct Hbg) sample—(Ct Telomere − Ct Hbg) reference DNA Samples from each participant will be assayed on the 96-well plate. There will be random distribution between plates, but ensuring that the age, sex, and group allocation of participants (intervention vs. control) were similar across plates. T. Separate reactions for telomere and single copy reference gene Hbg reaction will be carried out in paired 96-well plates. Reactions will be performed with a 7500 q Real-Time PCR Detection System (applied bioscience). The melt-curve analysis will be carried out at the end of the run to ensure specific primer binding. All assays were performed blinded to the study patients’ characteristics and clinical data.

### Cardinal markers of aging panel

Levels of insulin, Insulin-like growth factor-1 (IGF-1), cystatin, Growth differentiation factor (GDF-15), Natriuretic Peptide (NT proBNP), Interlueikin-6 (IL-6), Tumour Necrosis Factor—Receptor1 (TNF-R1) will be assessed by commercial Enzyme linked immunosorbent (R&D systems).

### Physical performance

Grip Strength will be assessed with a hydraulic grip strength dynamometer (Jamar Baseline® measurements; Fabrication Enterprises Inc, Elmsford, NY, USA). The average of two trials for dominant hand will be used. Predictive validity of hand grip strength has been shown previously for both disability and mortality [36]. Berg balance scale will be used to measure balance [37]. Gait speed will be assessed by self-paced 15-foot walk test following the procedures of the Fried Frailty Index 3 [38]. Functionality through Activities of daily living [39].

### Cognitive performance

Under cognitive function, processing speed will be assessed by Digit symbol substitution task (DSST) which consists of filling as many empty boxes as possible with a symbol matching each number [40]. Time is 90 s, and the total score is subtraction of the number of errors from correct matches.

## Statistical analyses

### Sample size

Total of 166 participants are planned to recruit for yBioAge which is estimated by g power software using a moderate effect size of f = 0.25 and a power of 80% (for alpha = 0.05), often recommended as appropriate power in behavioural research [41] the power analysis yielded a sample size of 128 participants. Allowing for 30% attrition in one year, we planned to recruit 38 participants per treatment condition to maximize power.

Baseline characteristics of participants will be summarised using mean and SD or median and IQR for continuous variables and frequency and percentage for categorical variables. The primary outcome analyses will observe intention-to-treat principle. Missing data will be replaced using Multivariate Imputation by Chained Equations in R studio software. Sensitivity analyses will be performed. Log binomial models will be estimated by generalised estimating equations. Mixed-effects linear regression models will be used. We will include outcomes at baseline, 26^th^ week and at the end of 52^nd^ week. All analyses will be performed in IBM SPSS, V.26 and in R (V.76). We will employ a statistical threshold of α = 0.05.

### Patient and public involvement

Study participants will be involved at the time of recruitment and once they meet the eligibility criteria, written informed consents will be obtained after explaining them their roles in study and procedures of trail by research assistants. Participation in the research will not result in any costs or burdens, and the intervention will be offered free of charge.

### Adherence and attrition

Overall adherence will be calculated by the attendance of participants in yoga sessions. Individual participants attendance rates will be calculated by dividing the number of yoga sessions attended by the total number of sessions conducted. Attrition rates, defined as the number of individuals who dropped out of the study divided by the total number of enrolled participants, will also be analysed for each group. Cited reasons for study drop out will then be summarized.

### Assessment of harms

All eligible candidates will be screened by trained research assistants to eliminate those with health-related problems that might disturb their participation. Personal meeting with the trainer after each session will be maintained throughout and feedback forms will be obtained on monthly basis by the research staff to ensure the safety and elimination of harm if any.

### Data management

A research study monitor will follow the progress of the study, and will also ensure that the participant's rights and well-being are upheld. He/she will also ensure that the protocol, ethical requirements, standards, and regulations are adhered to, that the data obtained is appropriately recorded. One of the research staff will monitor the coding, security, and storage of data. Furthermore, he or she will ensure correct entry of data values twice.

## Discussion

The fact that the extension of life has not been balanced by an equivalent lengthening of healthy life is now pretty obvious. The only sure-fire way to attain "Healthy Aging" is by carrying out "geroscience-guided" trials on human beings. It has not, however, received the proper scientific consideration due to a lack of credibility as a sign that requires intervention, despite the fact that it has been acknowledged that aging poses a large disease burden [14]. The dearth of well-planned, sizable geriatric clinical trials suggests that the need to cure or prevent aging has not received enough attention. Development and execution of anti-ageing medicines or interventions are essential for extending the "healthy" life span given the population's accelerated demographic aging transition.

Despite their high predictive capacity for mortality and age-associated dysfunctions, the BA-based composite markers have not been explored thoroughly as study outcomes of geriatric trials. The proposed work will be the first to evaluate the potential of yoga-based intervention to modulate algorithm-based BA estimates. These variables could be easily assessed compared to epigenetic methylation-based biological clocks, and have.

Physical activity has been reported to have a significant influence on ageing status [9]. There is substantial literature available on the efficacy of yoga on varied physiological functions [respiratory, metabolism, immune] that represent the hallmarks of physiological decline during ageing. Cost-effectiveness and other features of yoga, such as home practice, social interaction, and relaxation aspects, add to its acceptance in older adults. There are few important reports on the feasibility of yoga intervention in Yoga is a safe and cost-effective mind–body practice. Physical activity has been reported to have a significant influence on aging status [9]. There is substantial literature available on the efficacy of yoga on varied physiological functions [respiratory, metabolism, immune] that represent the hallmarks of physiological decline during ageing. Cost-effectiveness and other features of yoga, such as home practice, social interaction, and relaxation aspects, add to its acceptance in older adults. There are few important reports on the feasibility of yoga intervention in elderly population. Despite the growing body of research suggesting the acceptability of yoga-based interventions by older adults [16] to date, there has been no Yoga-based intervention trial reported to target “Biological Aging”. yBioAge would aid in catalysing interest amongst gerontologists to include mind–body interventions for Gero-science guided clinical research. The findings of this study could be a value addition to the gerontological scientific community given the safety and cost effectiveness of these interventions at par with calorie and dietary restrictions.

## Data Availability

As the paper relates to a study protocol, data sharing is not applicable as no datasets generated and/or analysed for yBioAge. However, after completion of the study, individual participant data that underlie the results will be reported in a peer-reviewed publication after de-identification in the form of an appendix. Hence, we feel that data sharing statement will be applicable in the manuscript that will arise from the proposed study. Primary results and datasets will be available from the corresponding author on reasonable request to the researchers who provide a methodologically sound proposal.
